# Perforated Duodenal Ulcer in a Young Nepalese Girl: An Infrequent Diagnosis for Age

**DOI:** 10.1155/2021/6304309

**Published:** 2021-11-18

**Authors:** Ashish Lal Shrestha, Anusha Shrestha

**Affiliations:** ^1^Department of Pediatric Surgery, Kathmandu Medical College and Teaching Hospital, Sinamangal, Kathmandu, Nepal; ^2^Department of Pediatrics, Grande International Hospital, Tokha Road, Kathmandu, Nepal

## Abstract

Perforated duodenal ulcer (PDU) is exceedingly uncommon in children. In a child with acute abdomen and pneumoperitoneum, an appendiceal etiology is more often suspected as a likely cause. Failure or delay to diagnose a PDU can result in significant morbidity and even mortality. We report a case of acute abdomen in a girl with PDU with a successful outcome. A 12-year-old school girl presented to emergency room (ER) with acute generalized abdominal pain for 2 days. Clinical examination revealed florid peritonitis, and abdominal radiographs showed free peritoneal air. At emergency laparotomy, PDU was noted with general peritoneal contamination. Omental patch repair and continued supportive care resulted in gradual improvement. PDU is an uncommon cause of peritonitis in children and poses significant challenges in management. Strong suspicion and prompt appropriate intervention is necessary to avoid untoward outcomes.

## 1. Introduction

Peptic ulcer disease (PUD) in children is unusual. Existing literature reporting a prevalence of 8.1% from Europe and incidence of 1.55 annual cases from India makes it an uncommon diagnosis [1, 2]. Furthermore, even rarer are the complications of PUD such as perforation and hemorrhage. Radiological pneumoperitoneum with peritoneal signs in a child should arouse a suspicion of PDU eventhough appendicitis is considered the commonest cause of surgical abdomen in children.

### 1.1. Case History Presentation

**Figure 1:**
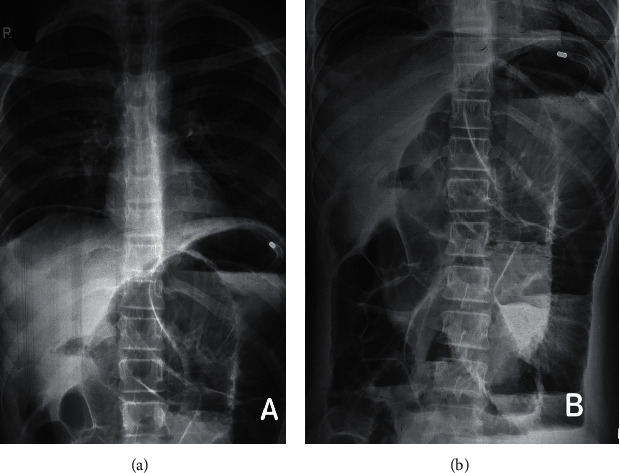
Free subdiaphragmatic extr luminal air and distended small bowel shadow in erect chest X-ray (a) and supine abdominal X-ray (b).

A 12-year-old previously healthy girl had presented to the ER with complaints of central abdominal pain for 3 days that had become generalized over 2 days. This was associated with multiple episodes of bilious vomiting and abdominal bloating. She was not receiving any medications and did not have history of fever or trauma. On examination, she was dehydrated, ill, and toxic looking and in obvious discomfort. She was tachycardiac but normotensive and afebrile. Abdomen was distended and guarded. There was generalized tenderness, board like rigidity, and absent bowel sounds. Her hemogram showed leucopenia (total leukocyte count: 1480 cells/Cu.mm and differential leukocyte count: polymorphs: 72%, lymphocytes: 21%, eosinophils: 1%, and monocytes: 6%) and elevated C-reactive protein: >90 mg/l. Her blood group was O positive. Biochemical tests showed mild hyponatremia (Na+: 130 mmol/l) and prerenal azotemia (urea: 75 mg/dl and creatinine: 1.2 mg/dl). Abdominal radiograph revealed free air under the diaphragm with gaseous generalized bowel distension as shown in Figure 1.

**Figure 2:**
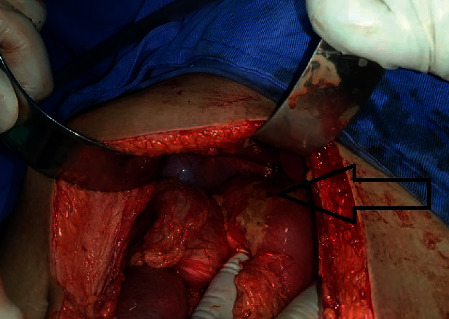
D1 perforation at laparotomy.

A working diagnosis of hollow viscous perforation peritonitis in prerenal azotemia and evolving sepsis was made. Following fluid resuscitation and first dose of intravenous broad spectrum antibiotics, laparotomy was performed. The findings were of generalized peritoneal contamination secondary to a perforated ulcer measuring 2 × 3 mm over the anterior surface of D1 as shown in Figure 2. Graham's omental patch closure of the perforation with feeding jejunostomy (FJ) and peritoneal irrigation was done.

Postoperatively, she was monitored in pediatric intensive care unit with continuation of supportive care. Nasogastric decompression, intravenous fluids, and antibiotics were continued. After 48 hours, FJ feeds were commenced and gradually upgraded. After resolution of the ileus, as suggested by lowering and clearing Ryle's tube aspirates and passage of flatus, nasogastric decompression was discontinued; oral feeds were started, and this could be achieved by day 5. Following this, oral feeds were gradually increased after removal of Ryle's tube. On tolerating oral feeds, FJ was clamped and left in situ.

**Figure 3:**
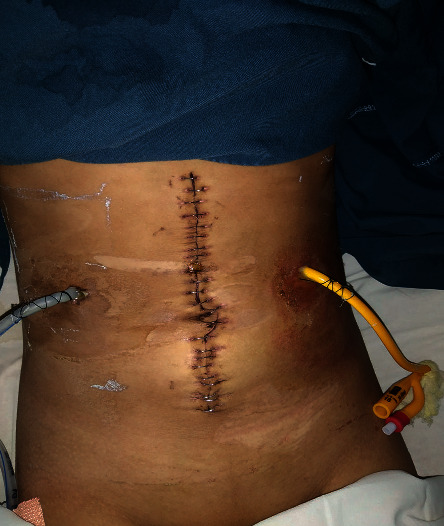
Subhepatic drain (right) and feeding jejunostomy tube (left) at follow-up.

She had a slow but a steady recovery and was discharged on day 11 with FJ as shown in Figure 3. At follow-up a week later in OPD, skin clips and FJ were removed. Tests for *Helicobacter pylori* infection were negative and serum gastrin levels were normal, and therefore, after a total of 2 weeks, proton pump inhibitors were discontinued. At 1 year follow-up, she continued to do well.

## 2. Discussion

Pediatric acute abdomen, often limited by difficulties in history taking and nonspecific findings on physical examination, poses a significant challenge to the clinician. When presented with peritoneal signs and radiographic findings of free subdiaphragmatic air, perforated appendiceal pathology is commonly suspected owing to its relative likelihood [3, 4]. However, PDU can also present with similar findings.

PDU, the commoner of peptic perforation, is nevertheless an uncommon complication of PUD which in itself is an infrequent diagnosis in children [1, 5–7]. The primary factors associated with PUD are blood group O, *H. pylori* infection, sickle cell disease, and Zollinger–Ellison syndrome [8]. Similarly, secondary factors implicated are medications (nonsteroidal anti-inflammatory drugs or steroids), severe systemic illness, neurotrauma, and burns [1, 2, 6, 9]. Apart from these, unusual pathologies such as malaria, gastroenteritis, meningitis, and even lymphoma have been reported in causation of PDU [3, 10]. With our patient, the only possible link that could be established was blood group O.

Irrespective of the known associations, the most crucial factor that could potentially change the outcome seems to be “timely intervention.” Operative delay beyond 12 hours, age, female gender, and larger perforations all in the past seem to have contributed to a poorer outcome [1, 11]. For an early diagnosis, however, a high index of clinical suspicion is necessary. This when aided with plain radiograph showing free subdiaphragmatic air clenches the diagnosis, although some studies prefer the use of computed tomography for an early and accurate detection of pneumoperitoneum [1].

The treatment is surgical repair which can be achieved either with laparotomy or laparoscopy depending upon the availability of local expertise and level of peritoneal contamination. A buttressed closure of the PDU with a tongue of vascularised omentum is a time tested method with reasonable success. In view of delayed presentation beyond 48 hours and anticipated ileus with our patient, a FJ was also performed and later used for timely commencement of enteral feeds. However, with relative paucity of studies determining the role of acid lowering procedures in children, it was not performed [3].

## 3. Conclusion

PUD is an uncommon diagnosis in children with its complicated presentations being further rare. When a child with acute abdomen is met with free intraabdominal air on radiographs, PDU should be ruled out as a possibility. Timely surgical intervention preceded by clinical suspicion is necessary to treat PDU and avoid the dreaded complications.

## Data Availability

The data used to support the findings of this study are available from the corresponding author upon request.

## Abbreviations

PDU: Perforated duodenal ulcer
PUD: Peptic ulcer disease
ER: Emergency room
FJ: Feeding jejunostomy.
